# 

**Published:** 1891-09-19

**Authors:** 


### NOTICE TO CORRESPONDENTS.

All MS., Letters, Books for Review, and other matters intended for the Editor should be addressed The Editor, The Lodge, Porchester Square, London, W.

The Editor cannot undertake to return rejected M.S., even when accompanied by stamped directed envelope.

**Notice to Advertisers and Subscribers.**

All Advertisements, Orders for Copies of The Hospital, and Business Communications should be addressed to The Publisher, *not to the Editor*, The Hospital, 140, Strand, W.C.

The Cost of Subscription, post free, is as follows:—Per week, 1½d.; per quarter, 1s. 8d.; per half-year, 3s. 3d.; per annum, 6s. 6d. Foreign:—Per annum, 8s. 8d.; payable, in all cases, in advance.

**NOTICE.**—The Index for Vol. IX. (ending March, 1891) is on sale at the Office of this Journal, price 2½d., post free.

### GENERAL CHARITIES.

[This Column is devoted to an account of work of charitable institutions other than Medical Charities. The Editor will be glad to receive reports or news of work at 140, Strand. *Philanthropy* should be plainly written on the envelope.]

There are now upwards of six hundred and eighty preventive and reformatory institutions affiliated to the **REFORMATORY AND REFUGE UNION**, which was founded some thirty-five years ago. The chief aim of the Union is to reclaim and elevate the neglected and criminal classes, to provide a central association for reformatories, refuges, industrial schools, and other voluntary institutions of a similar nature; by means of the Children's Aid Fund to assist in the rescue of destitute and neglected children, and to contribute towards their support in homes affiliated to the Union. Affiliated institutions are also assisted by grants of money, made after inspection by the Council. The Union also includes within its scope the promotion of the efficiency of the education and training given in these various institutions, and the collection and diffusion of information as to their work. The Institutions which are classified as child-saving provide food and shelter and industrial training to 45,000 children. The voluntary missionary agencies of the Union in London alone, for assisting and co-operating with the child-saving Institutions, deal with nearly 6,000 children annually. The Council, in their latest report, while fully recognising that the children now in industrial schools have many of them been rescued from the vilest haunts, protest against the assumption that has recently been put forward, that these children are still a portion of the "submerged tenth." We agree with the Council in their view that the most hopeful means, in fact the only means likely to meet with lasting success, of working for the permanent benefit of mankind, is to get at the rising generation while still young and capable of being moulded to higher and better things. If you cannot rescue children, depend upon it you need waste no energy on adults. Much of the amount of money spent by the State on gaols, or in other words, on shutting the stable door when the steed has gone, might well be employed in a more fruitful manner. If only a tithe of this money had from the first been devoted to the promotion of institutions which really rescue and reform children, the criminal population of the country would be much fewer in numbers than it is now, and a large proportion of the neglected children of the metropolis who now go to swell the criminal ranks would have been brought up to earn an honest livelihood, and to have been a benefit instead of an expense to the country. It is the custom of the Union to send an officer to attend the metropolitan police courts, with instructions to deal with all children needing the society's help, who are sent to various homes and industrial schools throughout the country. Emigration work is also carried on by the Union, which sent twenty-six children to Winnipeg last year, and made arrangements early in the present year for two more parties to go out. In order to promote co-operation amongst the managers and workers of the numerous affiliated institutions, periodical gatherings are held at certain centres for conference and social intercourse. Evening meetings are held quarterly in the metropolis, and a conference lasting three days is held once in three years. During the past year the Highbury Truant School, the Montague House Home for Inebriates, Amesbury House Home for Inebriates, and Southwell House Temporary Home, have been certified or affiliated to the Union. A branch of the Society's work which must commend itself to all is the effort which it is now making to arrive at a right solution of the problem how best to provide for the welfare of the young, who, though mentally weak, are not so imbecile as to be eligible for entry into any existing hospitals or infirmaries. This question has been occupying the attention of a special committee. Another matter which has also engaged the serious attention of the Council is the number of persons repeatedly committed for drunkenness. That the present method, or rather want of method, of dealing with habitual drunkards is a failure there is no doubt, and it would be to the interest of society, as well as of these unfortunate people themselves, if some practical method of treatment could be applied to them. On this point most people are agreed, but we are not aware that the Society is in a position to make any suggestions likely to lead to a practical and lasting result. Changes for the better, we are glad to say, have during recent years taken place in the management of reformatory and industrial schools. The unpopularity of the system of necessarily submitting children to a term of imprisonment previous to their admission to a reformatory school has steadily increased. As has been pointed out by Her Majesty's Inspector, it is well to send youths to reformatories for first offences which indicate a criminal tendency, but the practice of sending children of this class to industrial schools before they are allowed even a chance of breaking the law will be found more economical, and will exercise a more deterring effect on the criminal statistics of the country than any system of reformatory discipline or deterrent prison sentence. The accommodation in the schools has also been much improved, and the school teaching is undoubtedly more efficient. For instance, in former years it was only too common for youths leaving the schools to have no knowledge beyond that which is available in the unskilled labour market, and it was a step in the right direction to substitute practical carpentering for wood chopping. Much is still to be desired in the form of increased technical instruction and more thoroughness in the industrial training. Contributions in aid of the work of the Union should be sent to the Secretary, 32, Charing Cross, S.W.

### SCRAPS AND GLEANINGS.

THE Congress of Orientalists decided that rheumatism and microbes were both existent in pre-historic ages.

DR. PAGE, of Newcastle, has offered £1,000, on behalf of a patient of his, towards the proposed new Infirmary for Newcastle.

FATHER GORMAN and Mr. Coope have, we are glad to hear, been elected on to the House Committee of the London Hospital.

THE charges of maltreatment at the Coventry Hospital have entirely collapsed, and a vote of confidence in Dr. Parsons has been passed.

AT a preliminary public meeting held in Stourbridge, a committee was appointed in furtherance of a projected scheme to establish a cottage hospital.

THE Duchess of Albany has consented to open the new buildings forming the extension of the Printers' Almshouses at Wood Green, N., at an early date in October.

IT is proposed to transfer the management of the Woodlands Convalescent Home to the Bradford Infirmary authorities, in order to extend its sphere of usefulness.

ON Saturday week last the annual hospital demonstration took place at Knottingley and in spite of much rain, passed off fairly satisfactorily. A collection of over £60 was made.

THE Board of Directors of the Aberdeen Lunatic Asylum have at present under consideration certain proposals for the improvement and extension of the institution, which, if carried out, may cost £40,000 to £50,000.

DR. ALEXANDER MILES, of Edinburgh, was lately awarded a gold medal for his degree theses. Amongst the many students lately turned out by the Northern University, Dr. Miles gives great promise of future distinction.

A PORTRAIT of Lady Shaftesbury is to be placed in the Board-room of the Belfast Royal Infirmary, in commemoration of her generous gift of the site and premises of the institution to the Corporation at a nominal rent of 2s. 6d. per annum.

SUFFICIENT money has been collected to justify the establishment of a home for women suffering from incurable forms of cancer, and the committee are now seeking a house. It is hoped that some habitation may be offered for the purpose.

IT is to be regretted that the Bromwich District Hospital, which has done good work during the past year, should be suffering from want of funds. Three years ago the cost per bed was £51, it has now risen to £64. Surely there must be some mismanagement somewhere.

A SERIOUS outbreak of smallpox is reported in various districts of Leeds. The largest number of cases have occurred at Hunslet; but, so far, there have been no fatal results. Every precaution is being taken by the sanitary authorities to prevent the spread of the epidemic.

AN inquiry into the hospital dietry by the Liskeard Guardians seems not to have been made too soon. It seems an extraordinary thing that the Board should not have been aware that the infirmary diet was the same as that of adult paupers of the Union to which it appertains.

A DISPUTE has arisen at the Salisbury Infirmary respecting the election of the house surgeon. Mr. Wilkes has been elected, but Mr. Hogarth, the unsuccessful candidate, appeals, on the ground that some of the electors left the meeting before their votes were finally recorded.

THE Prince of Wales has appointed the Earl of Limerick Director of the Ambulance Department of the Order of the Hospital of St. John of Jerusalem in England. Since the opening of the last winter session on October 1st over 28,000 first aid and nursing certificates have been issued.

THE annual report of the Worthing Infirmary shows that the work during the year has been exceptionally heavy. A slight falling off in the receipts is shown, and as it is found necessary to increase the nursing staff special efforts towards an increase of income will be essential.

AT the annual court of the Governors of the Salisbury Infirmary it was stated that an extension of the institution is contemplated. Eight hundred and eighty-three in-patients have been admitted during the year, and thirty-five have been sent to the Herbert Convalescent Home at Bournemouth.

THE cottage hospital now opened at Coleraine, Ireland, was commenced four years ago, and owes its success mainly to the exertions of Miss Macausland, of Garvagh. The institution has been erected by Mr. Douglas, a local builder, and possesses excellent accommodation, with large and airy wards.

THE Hospital Saturday Collections for the Portsmouth Hospital took place on Saturday week last. The arrangements were similar to those of last year. The amount received from boxes will not be made known for some months, as they remain out for some time. The boys' collections realised £43 1s. 1¾d., against £26 12s. 5¼d. last year.

THE City of Edinburgh's Convalescent Home, Campie House, Musselburgh, was the first Corporation Convalescent Home established in Scotland. With the view of making the institution better known, the Public Health Committee are to hold a garden party at the house, to which the members of the Council and their friends, as well as a number of others interested in such work, in all about two hundred, are to be invited.

THE twenty-second anniversary of the opening of the West of Scotland Convalescent Seaside Home, Dunoon, was celebrated recently. The statistics of the home show that the desire to benefit those who sought its rest and care have been wonderfully well fulfilled. During the past year about 3,300 persons have benefited by a short stay in the institution. The management is all that can be desired, and the institution is efficiently and economically administered.

AT an inquest in St. Pancras, concerning the death of a lady of independent means, it was stated that at Llandudno she slipped in stepping from her carriage and injured her leg. The doctor who attended her had made no reference in his certificate to the accident, and the coroner said that if he was really aware of it he could be fined £40 for the suppression of this essential particular, and the registrar who accepted such certificate was liable to a like penalty.

### The Student of Medicine.

[All communications for this Supplement should be addressed to The Editor of The Hospital, at 140, Strand, with the words, "Students' Column," written in the left hand top corner of the envelope.]

### APPOINTMENTS.

At the Monsall Fever Hospital, Manchester, Edward Mansfield Brockbank, M.B., Ch.B., Vict., and W. B. Warrington have been appointed house physicians; Richard Clegg, L.R.C.P., M.R.C.S., William James Howarth, L.R.C.P., L.R.C.S., Edin., house surgeons, and Alexander Johnson, M.D., M.B., C.M., Glas., resident medical officer. At the General Infirmary at Gloucester and Gloucester Eye Institution, John Law Adam, M.B., C.M., Aberd., has been appointed house surgeon. At the County and Borough Infirmary, Carmarthen, Sidney Lucy, M.R.C.S., L.R.C.P., has been appointed house surgeon. At the Coventry and Warwickshire Hospital, George G. Parsons, L.R.C.P., M.R.C.S., L.S.A., has been appointed house surgeon. At the Stockport Infirmary, Henry Reynell Bellamy, L.R.C.P., L.R.C.S., Edin., has been appointed house surgeon. At the Leeds General Infirmary, H. E. Tomlinson, L.S.A., has been appointed second house physician. At the Royal Victoria Hospital, Bournemouth, Gilbert Wickham, B.A., M.B., B.C., Cantab., has been appointed house surgeon and secretary.

### VACANCIES.

The following vacancies are announced this week, the amount of salary in each case being given after the name of the institution:

### Resident Medical Officers.

Bethlem Hospital, S.E., Two Resident Clinical Assistants—Board, &c.
City of London Hospital for Diseases of the Chest, Victoria Park, E., House Physician—Board, &c.
Eastern Counties Asylum for Idiots, Colchester, Resident Medical Attendant—Salary £100 and board, &c.
Islington Dispensary, 303, Upper Street, Islington, Resident Medical Officer—Salary £200 and cab hire.
Kent County Asylum, Barming Heath, Maidstone, Senior Assistant Medical Officer—Salary £250.
Royal National Hospital for Consumption, Ventnor, Isle of Wight, Resident Medical Officer.
West London Hospital, House Physician and House Surgeon.

### Non-Resident Medical Officers.

Charing Cross Hospital, Surgical Registrar.
City Dispensary, Watling Street, E.C., Surgeon.
Dental Hospital of London, Leicester Square, Anæsthetist and Assistant Anæsthetist.

### SCHOLARSHIPS AND PRIZES.

### University of Aberdeen—Summer Session, 1891.

EMBRYOLOGY AND VISCERAL ANATOMY.

First Class Certificate of Merit.—A. H. Lister, B.A., Essex (prize), 98; J. M. H. Macleod, M.A., Dundee, 87; W. C. Hossack, Banff, 85·5.

Second Class Certificates of Merit.—J. H. Rewe, Cornwall, 72·5; A. P. Rust, Peterculter, [illegible]7; J. S. Marr, Drumoak, 62·5; L. Adam, Banchory, 61·5.

EMBRYOLOGY (Advanced Students).

First Class Certificates of Merit.—A. W. Mackintosh, M.A., Aberdeen (prize), 91·5; A. Brown, M.A., B.S., Ordiquhill (prize), 89.

Second Class Certificates of Merit.—Simon J. C. Fraser, Dooar, India, and William Thomson, M.A., Turriff—equal, 72; John H. Goodliffe, Nottingham, 69; P. M. Muttukumaru, Colombo, and G. J. A. Watson, M.A., Strichen—equal, 60·5.

OSTEOLOGY.

First Class Certificates of Merit.—A. S. Gage, M.A., Aberdeen (prize), 89; A. Fenton, M.A., Ellon (prize), W. G. Grant, Elgin (prize), and G. B. Scott, Peterculter (prize), equal 87; J. R. Smith, Ellon, 80·5; W. M. Philip, Skene 78·5; Alexander Robb, M.A., Aberlour, 77; John Skinner, Insch; William Murray, M.A., Peterhead, and James S. Warrack, Stranraer—equal, 7[illegible].

Second Class Certificates of Merit.—E. K. G[illegible]wn, Derby, 73·5; M. MacBean, Kingussie, and G. A. Reid, Beauly—equal, 71; H. Duffus, M.A., New Pitsligo, 68; R. Lindsay, Tomintoul, 67·5; G. Mitchell, Keith, 66; A. H. Mackie, M.A., Aberdeen, 65·5; A. M. Malcolm, Aberdeen, and A. Thompson, Elgin—equal, 62·5; P. H. Bannister, Havant, and John W. Myers, Leeds—equal, 62; T. D. Cumming, Aberdeen, and J. W. Milne, New Deer—equal, 61·5; J. A. Milne, M.A., Aberdeen, and A. Sharp, M.A., Cultercullen—equal, 61; A. Mowat, Invergordon, 60·5.

### PASS LISTS.

### Society of Apothecaries of London.

The following candidates have passed the examination in the subjects indicated:—

Surgery.—J. Fearnley, Yorkshire College, Leeds; A. S. Phillips, St. Thomas's Hospital; T. Prescott, King's College; P. Mackin, Glasgow; E. R. Sims, Leeds and Glasgow; H. S. Smith, Toronto; W. R. Thomas, London Hospital and Montreal; S. B. Williams, St. Mary's Hospital.

Medicine, Forensic Medicine and Midwifery.—J. Crooks, Toronto; J. Fearnley, Yorkshire College, Leeds; P. Mackin, Glasgow; J. J. Mooney, Manchester; C. K. Moseley, St. Bartholomew's Hospital; W. Overend, St. Mary's Hospital; S. C. Smith, Middlesex Hospital; C. H. C. Viscik, University College.

Medicine and Midwifery.—A. Paling and G. J. Rutherford, Middlesex Hospital; J. D. H. Smyth, St. Mary's Hospital.

Forensic Medicine and Midwifery.—G. Grace, Bristol.

Medicine.—H. E. Pittway and W. A. Ward, Middlesex Hospital.

Midwifery.—E. E. Frazer, Guy's Hospital; P. T. Naden, Birmingham.

Forensic Medicine.—J. F. Brown, Toronto; B. F. Parish, St. Mary's Hospital.

To Messrs. Brown, Fearnley, Mackin, Parish, Phillips, and S. C. Smith was granted the Diploma of the Society, entitling them to practice Medicine, Surgery, and Midwifery.

### University of Glasgow.

The following is the list of those on whom medical degrees were conferred by this University at the graduation ceremony on the 30th ult.:

Doctors of Medicine.—*With Honours*: Hugh Rhodes, M.B., C.M., England. *With Commendation*: Gilbert Alex. Bannatyne, M.B., C.M.; Andrew Nicholson M'Gregor, M.B., C.M.; and Andrew Maitland Ramsay, M.B., C.M., all of Scotland. *Ordinary Degree*: Alexander Charles Farquharson, M.B., C.M.; Hugh Girvan, M.B., C.M.; Robert Lachlan Pinkerton, M.A., M.B., C.M.; William Robertson, M.B., C.M.; James Stevenson, M.B., C.M.; and John Stewart, M.B., C.M., all of Scotland.

Bachelors of Medicine and Masters in Surgery.—*Honours*: *Hugh Galt and Robt. C. Robertson, M.A., both of Scotland. *High Commendation*: John William Logie, Daniel Macpherson Taylor, M.A., George Clark Stewart, John M'Gregor, Daniel M'Kenzie, Niel Campbell, and John Gilmour, all of Scotland. *Commendation*: Robert Wm. Nairn, Brownlow Riddell, John Frew, James Todd, David Smith, Wm. James M'Kendrick, B.Sc., and John Yuill, all of Scotland; Robert M'Ghie, and George Mervyn Sydenham, England. *Ordinary Degrees*: James Aitken, William Anderson, John Fulton Barr, B.Sc., John Adam Boyd, Alexander Campbell, David Christie, Samuel Cockburn, David Coutts, John Alex. Creighton, William Crichton, Hugh Colligan Donald, Wm. Bertram Ochiltree Ferguson, David Wyllie Girvan, Andrew Goldie, John Angus Green, Richard Hamilton, Robert Home Henderson, Anthony Inglis, Robert James, Robert Robertson Kilpatrick, Peter Alexander Laird, Chas. Lavery, Matthew Lochhead, B.Sc., Robert Mortimer Malcolm, James Marr, Thomas Milbourne Martin, William Mason, John Wilson Mathie, John Miller, Robert Walker Moir, David Muir, John Kerr Muir, William Murray, Dugald Macdougall, Donald Neil Macfarlane, Neil Macintyre, Jas. Henderson Naismith, Gilbert Park, Hugh Hamilton Park, William Park (Beith), Robert Alexander Paton, Andrew Robertson, Archibald George Sanders, Arthur Thomas Scott, M.A., Robert Sharp, Robert Stobo, Charles Symington, Lewis Dunbar Temple, Ebenezer Turner, Wm. Thompson Merry Wallace, David Watson, William Watson, James Laurie White, Martin Whyte, James Allan Wilson, and Robert Wilson, all of Scotland; James Gordon Bain, Herbert Du Cane, William Doyle, and Alex. Lewis M'Leod, M.A., all of England; Peter Otho Watkin Browne, John David, John Harris Jones, and David Lloyd, all of Wales; Yousuf Hamis, Syria; Ernest Louis Marsh, Ireland; Chas. Hugh M'Ilraith, M.A., Holland. *Mr. Galt gains the Brunton Memorial Prize of £10, awarded to the most distinguished graduate in Medicine of the year.

### Royal Colleges of Physicians and Surgeons of Edinburgh and Faculty of Physicians and Surgeons of Glasgow.

At the July sittings of the Examiners, held in Glasgow, the following candidates passed:—

First Examination.—Of 47 candidates entered the following 28 passed: Alfred E. J. Ward, India; Hugh Mackay, Glasgow; Lydia Ann Leney, Sussex; John F. W. Ward, India; Marion Gilchrist, Bothwell; Alice L. L. Cumming, Renfrewshire; Marion C. Horner, co. Derry; Flora A. Holt, Yorkshire; W. H. Penrose, Cork; Elizabeth D. Lyness, co. Antrim; George Gibson, co. Cork; Thomas William Smyth, co. Cork; Charlotte R. Hodgins, co. Tipperary; Margaret C. Dewar, Ceylon; Amy C. Dewar, Ceylon; John H. Holmes, Llanidloes; Luke J. Quigley, King's County; Janet M. Kay, Castle-Douglas; David Walker, Glasgow; Anthony S. Rose, Morpeth; Joseph Tibbitts Kinver, Staff.; William L. Norwood, Ireland; William A. Reidy, Ireland; Ebenezer Allan, Scotland; Charles Peddie, Scotland; David Dinwoodie, Scotland; William Watson, Scotland; Charles E. Moody, Scotland.

Second Examination.—Of 59 candidates entered, the following 28 passed: George J. Willis, Queen's County; James J. Fanning, co. Tipperary; Joseph F. Campbell, Athlone; John Russell, Aberdeenshire, George E. Macleod, India; David Stephen, co. Down; William D. Anderson, London; William Owen, Carnarvon; Patrick J. O'Keeffe, Cork; John P. Roberts, Ruthin; Wm. C. Paterson, Wetherby; John Livingston, Glasgow; Lachlan Burges, Banffshire; John C. Lynd, Limavady; Percy T. Tolputt, Folkestone; Samuel M'Nair, co. Antrim; Henry W. Stewart, co. Tyrone; Henry R. Wolfe, Glasgow; Cuthbert Rutherford, Northumberland; James C. Hamilton, co. Tyrone; Patrick J. Murphy, Cork; William R. Thomson, Australia; Chas. R. Huxley, Kent; Herbert P. Weston, Manchester; Barclay Wiggins, Ayrshire; Albert Edward Downey, Glasgow; Wm. C. Alexander, Glasgow; John Clark, Glasgow.

Final Examinations.—Of 56 candidates entered, the following 18 passed and were admitted licentiates of the three Colleges: John C. Ferguson, Edgehill, Forres; Maurice Gepp, Chelmsford; Henry B. Ord, Darlington; David M'Crorie, Glasgow; Patrick Mackin, Glasgow; Francis Edward Williams, Edinburgh; Marshall Sutton, M.D., Edinburgh; William A. Gosford, Liverpool; Murdo Murchison, Edinburgh; John Lamont, Partick; Herbert Part, Wigan; Henry A. Holmes, Manchester; Thos. L. Wilson, Glasgow; Grace Rose Cadell, Edinburgh; Thomas G. Carr, Preston; James Wardrop, Glasgow; Wm. G. Scarth, Leeds; Joseph B. H. Beatty, London.

### Army Medical Service.

The following list has been issued of the successful candidates at the recent competitive examination for comissions in the medical staff of her Majesty's Army:—

| | Total number of Marks. | | Total number of Marks. |
|---|---|---|---|
| Pilcher, E. M. ... ... | 3300 | Alexander, J. D. ... ... | 2645 |
| Johnson, H. P. ... ... | 3165 | Holt, R. H. E. G. ... ... | 2640 |
| Beyts, W. G. ... ... | 2970 | Kelly, J. F. M. ... ... | 2635 |
| Stalkartt, H. A. ... | 2780 | Dunn, H. N. ... ... | 2620 |
| Morphew, E. M. ... | 2710 | Hughes, G. E. ... ... | 2620 |
| Fleming, C. C. ... ... | 2700 | Martin, C. B. ... ... | 2610 |
| Tyacke, N. ... ... | 2700 | Withers, S. H. ... ... | 2600 |
| Mitchell, L. A. ... ... | 2690 | Buchanan, G. J. ... ... | 2580 |
| Lawson, C. B. ... ... | 2680 | Crawford, G. S. ... ... | 2550 |
| Anderson, E. C. ... | 2670 | Hennessy, J. ... ... | 2550 |

